# Interobserver agreement of skeletal muscle mass measurement on head and neck CT imaging at the level of the third cervical vertebra

**DOI:** 10.1007/s00405-019-05307-w

**Published:** 2019-01-28

**Authors:** S. I. Bril, A. W. Wendrich, J. E. Swartz, I. Wegner, F. Pameijer, E. J. Smid, G. H. Bol, A. J. Pothen, R. de Bree

**Affiliations:** 1 Department of Head and Neck Surgical Oncology, University Medical Center Utrecht, House Postal Number Q.04.5.300, Heidelberglaan 100, PO BOX 85500, 3584 CX Utrecht, The Netherlands; 2 Department of Otorhinolaryngology - Head and Neck Surgery, University Medical Center Utrecht, Heidelberglaan 100, 3584 CX Utrecht, The Netherlands; 3 Brain Center Rudolf Magnus, University Medical Center Utrecht, Heidelberglaan 100, 3584 CX Utrecht, The Netherlands; 4 Department of Radiology, University Medical Center Utrecht, Heidelberglaan 100, 3584 CX Utrecht, The Netherlands; 5 Department of Radiation Oncology, University Medical Center Utrecht, Heidelberglaan 100, 3584 CX Utrecht, The Netherlands

**Keywords:** Head and neck neoplasms, Sarcopenia, Body composition, Computed tomography, Observer variation

## Abstract

**Objectives:**

Skeletal muscle mass (SMM) is most often assessed in cancer patients on abdominal computed tomography (CT) imaging at the level of the third lumbar vertebra (L3). Abdominal CT imaging is not routinely performed in head and neck cancer (HNC) patients. Recently, a novel method to assess SMM on a single transversal CT slice at the level of the third cervical vertebra (C3) was published. The objective of this study was to assess the robustness of this novel C3 measurement method in terms of interobserver agreement.

**Patients and methods:**

Patients diagnosed with locally advanced head and neck squamous cell carcinoma (LA-HNSCC) at our center between 2007 and 2011 were evaluated. Fifty-four patients with were randomly selected for analysis. Six observers independently measured the cross-sectional muscle area (CSMA) at the level of C3 using a predefined, written protocol as instruction. Interobserver agreement was assessed using intraclass correlation coefficients (ICCs), a Bland–Altman plot and Fleiss’ kappa (κ).

**Results:**

The agreement in vertebra selection between all observers was excellent (Fleiss’ κ: 0.96). There was a substantial agreement between all observers in single slice selection (Fleiss’ κ: 0.61). For all CSMA measurements, ICCs were excellent (0.763–0.969; all *p* < 0.001). The Bland–Altman plot showed good agreement between measurements, with narrow limits of agreement.

**Conclusion:**

Interobserver agreement for SMM measurement at the level of C3 was excellent. Assessment of SMM at the level of C3 is easy and robust and can performed on routinely available imaging in HNC patients.

## Introduction

Body composition increasingly is a subject of interest in medical research. An abnormal body composition, such as a decreased lean body mass and/or increased adipose tissue mass, may have a profound influence on treatment outcome and (disease free) survival of patients with a variety of illnesses [1–3]. Skeletal muscle mass (SMM) is the largest component of lean body mass [4]. In cancer patients, a low SMM, sometimes referred to as sarcopenia, has specifically been associated with a higher incidence of chemotherapy-related toxicity, postoperative complications, longer hospital stay, increased healthcare-related expenditures, and lower disease free and overall survival [5–10]. This relationship has been shown in breast, colorectal, hepato-pancreatico-biliary, renal, and lung cancer, amongst others [11–15].

In cancer patients, SMM is most often assessed on abdominal computed tomography (CT) imaging at the level of the third lumbar vertebra (L3) [16]. This method is based on research using whole-body magnetic resonance imaging (MRI), in which has been shown that cross-sectional skeletal muscle area (CSMA) on a single transversal slice at the level of L3 is strongly correlated with total skeletal muscle volume as measured using whole-body MRI [17, 18]. The CSMA at the level of L3 is commonly normalized for stature, which results in the lumbar skeletal muscle index (lumbar SMI) [11]. This value is used as an indication of total SMM.

Abdominal CT imaging is often routinely performed in most types of cancer during diagnostic work-up and follow-up. In these patients, SMM measurement can be performed on abdominal CT imaging without the need for additional imaging or other diagnostics. However, abdominal CT imaging is not routinely performed in head and neck cancer (HNC) patients [19]. Recently, a novel method to assess SMM on a single transversal CT slice at the level of the third cervical vertebra (C3) was published [20]. Using this method, skeletal muscle mass is assessed measuring the CSMA of the paravertebral muscles and the sternocleidomastoid muscles at the level of the C3 vertebra. This method allows for evaluation of SMM in HNC patients on routinely performed imaging, in a similar manner as is used in patients with other types of cancer. This measurement method for SMM was recently used in three studies in head and neck cancer patients [21–23].

To be clinically useful, the C3 measurement method of SMM has to provide similar results when used by different observers. The aim of this study was to evaluate the interobserver agreement of SMM measurement at the level of C3. The robustness of the C3 measurement method was investigated in terms of the vertebra selection, the exact single slice selection, and the correspondence in CSMA measurements between all observers.

## Patients and methods

### Ethical approval

The design of this study was approved by the Medical Ethical Research Committee of our center (approval ID 14–544/C). All procedures in this study were in accordance with the ethical standards of the institutional and/or national research committee and with the 1964 Helsinki declaration and its later amendments or comparable ethical standards. All data were retrieved retrospectively. Measurements of SMM were performed on coded CT scans.

### Patients and study design

Patients diagnosed with locally advanced head and neck squamous cell carcinoma (LA-HNSCC) at the our center between 2007 and 2011 were evaluated for this study. Fifty-four patients with pre-treatment head and neck CT imaging were randomly selected for inclusion. Other parameters, including length and weight at the time of imaging, sex, age, tumor localization, and clinical TNM stage (7th edition), were retrospectively retrieved [24].

### Imaging protocol and analysis

All patients underwent contrast-enhanced CT scanning prior to radiation treatment on a Philips Brilliance iCT scanner (Philips Healthcare, Best, The Netherlands) using a standardized protocol for HNC patients. The imaging was performed in treatment position in a radiotherapy immobilization mask. Scanning parameters included slice thickness 1 mm with a 2 mm interslice gap.

### Measurement of SMM

Delineation of CSMA was manually performed using the Volumetool v.1.6.5 Research Software Package, designed in our center as an image evaluation, registration, and delineation system for radiotherapy planning [25]. Hounsfield Unit (HU) thresholds for skeletal muscle tissue were − 29 and + 150 HU; the area within these HU thresholds was defined as the CSMA. Delineation of CSMA was performed independently by six observers: one experienced head and neck radiologist, one experienced head and neck radiation oncologist, and four medically trained researchers from the departments of Head and Neck Surgical Oncology, and Otorhinolaryngology Head and Neck Surgery. A predefined, written protocol for single slice selection and CSMA was provided, as described in the recently published study by Swartz et al. [20]. In brief, the first slice when scrolling from caudal to cranial direction to show both transverse processes and the entire vertebral arc of the third cervical vertebra had to be selected. Contours of the paravertebral muscles (PVM) and both sternocleidomastoid (SCM) muscles were manually traced. This study was performed without a training data set, as to simulate the use of this measurement method as if it were adopted from an external research paper.

**Fig. 1:**
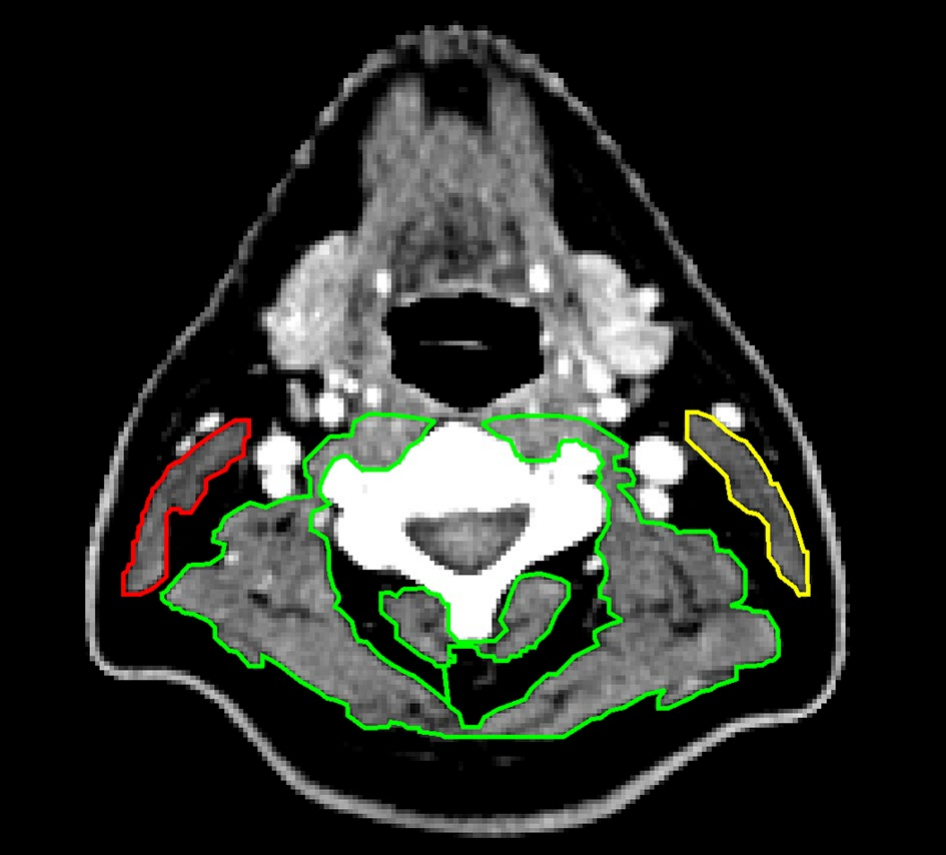
Example of delineation of skeletal muscle at the level of C3. The paravertebral muscles are delineation in green. The left sternocleidomastoid muscle is delineated in yellow, and the right sternocleidomastoid muscle in red

All observers independently selected the slice they deemed to be correct from the CT scan, and delineated CSMA. Observers were blinded to their own and each other’s results. After delineation of CSMA at C3 was finished by all observers, CSMA was automatically retrieved from Volumetool. Vertebra and single slice selection were investigated by reopening all scans after CSMA retrieval and analysis was finished; the selected vertebra and single slice location were noted. Total CSMA at C3 was calculated as the sum of the CSMA of the PVM and both SCM muscles. Figure 1 shows an example of CSMA delineation at the level of C3.

All statistical analyses were performed using the IBM SPSS Statistics version 21.0 software package (Chicago, Illinois, USA). A test for normality (Shapiro–Wilk test) was used to assess whether continuous variables were normally distributed. Continuous data are represented as mean ± standard deviation (SD). Categorical data are represented as a number and percentage of total. Differences in skeletal muscle area measurements between observers were calculated using a repeated measures ANOVA. Agreement between different observers was assessed by calculating Fleiss’ kappa (κ) and intraclass correlation coefficients (ICCs) using a two-way mixed single measures model with absolute agreement and. The κ values were graded as slight (0.01–0.20), fair (0.21–0.40), moderate (0.41–0.60), substantial (0.61–0.80), or almost perfect (0.81–0.99) agreement [26]. The ICCs were rated as poor (0.00–0.49), fair to good (0.50–0.74), and excellent (0.75–1.00) [27]. Bland–Altman plots were constructed to visualize agreement [28]. Results were considered statistically significant if the *p* value was less than 0.05.

## Results

### Patient characteristics

Baseline characteristics of the 54 included patients are shown in Table 1. Patients were predominantly male and mostly presented with lymph node positive, stage IV disease.

**Table 1:** Baseline patient characteristics

	Total *n* = 54
Sex	
Male	36 (66.7%)
Female	18 (33.3%)
Age	56.8 (7.3)
Weight	67.8 (13.2)
BMI	22.9 (3.8)
T-stage	
T1-2	13 (24.1%)
T3-4	41 (75.9%)
N-stage	
N0	5 (9.3%)
N +	49 (90.7%)
Clinical TNM stage^a^	
Stage III	5 (9.3%)
Stage IV	49 (90.7%)
Tumor site	
Oral cavity	6 (11.1%)
Nasopharyngeal	10 (18.5%)
Oropharyngeal	20 (37.0%)
Hypopharyngeal	8 (14.8%)
Laryngeal	5 (9.3%)
Other^b^	5 (9.3%)

Continuous data are represented as mean (SD). Categorical data are represented as number (% of total)

^a^Staged according to the AJCC Cancer Staging Manual 7th edition

^b^Other = sinus (*n* = 1), proximal esophagus (*n* = 2), and multiple (*n* = 2)

### Vertebra and single slice selection

Table 2 shows the results of the vertebra and single slice selection analysis. For the vertebra selection, the overall correspondence between all observers was near perfect (Fleiss’ κ: 0.96, *p* < 0.001). The correct vertebra (C3) was identified in almost all patients by all observers; in five patients, one observers chose a different vertebra, and in one patient, two observers chose a different vertebra. Either C2 or C4 was selected in these patients.

**Table 2:** Vertebra and single CT slice selection

	Vertebra (*n* = 54)	Slice (*n* = 54)
All observers same	49 (88.9%)	12 (22.2%)
One observer different	5 (9.1%)	16 (29.6%)
Two observers different	1 (1.9%)	12 (22.2%)
Three observers different	–	14 (25.9%)

Values shown as number (% of total)

There was more variation in exact single slice selection. The overall correspondence between all observers was substantial (Fleiss’ κ: 0.61, *p* < 0.001). In 22.2% of patients, all observers chose the same identical slice to delineate, while in the other 77.8%, at least one observer chose a different slice. In 79.3% of the cases, where a different slice from the majority was chosen, the different slices were located directly above or below the slice that the majority of observers chose.

### Cross-sectional skeletal muscle area measurements

**Fig. 2:**
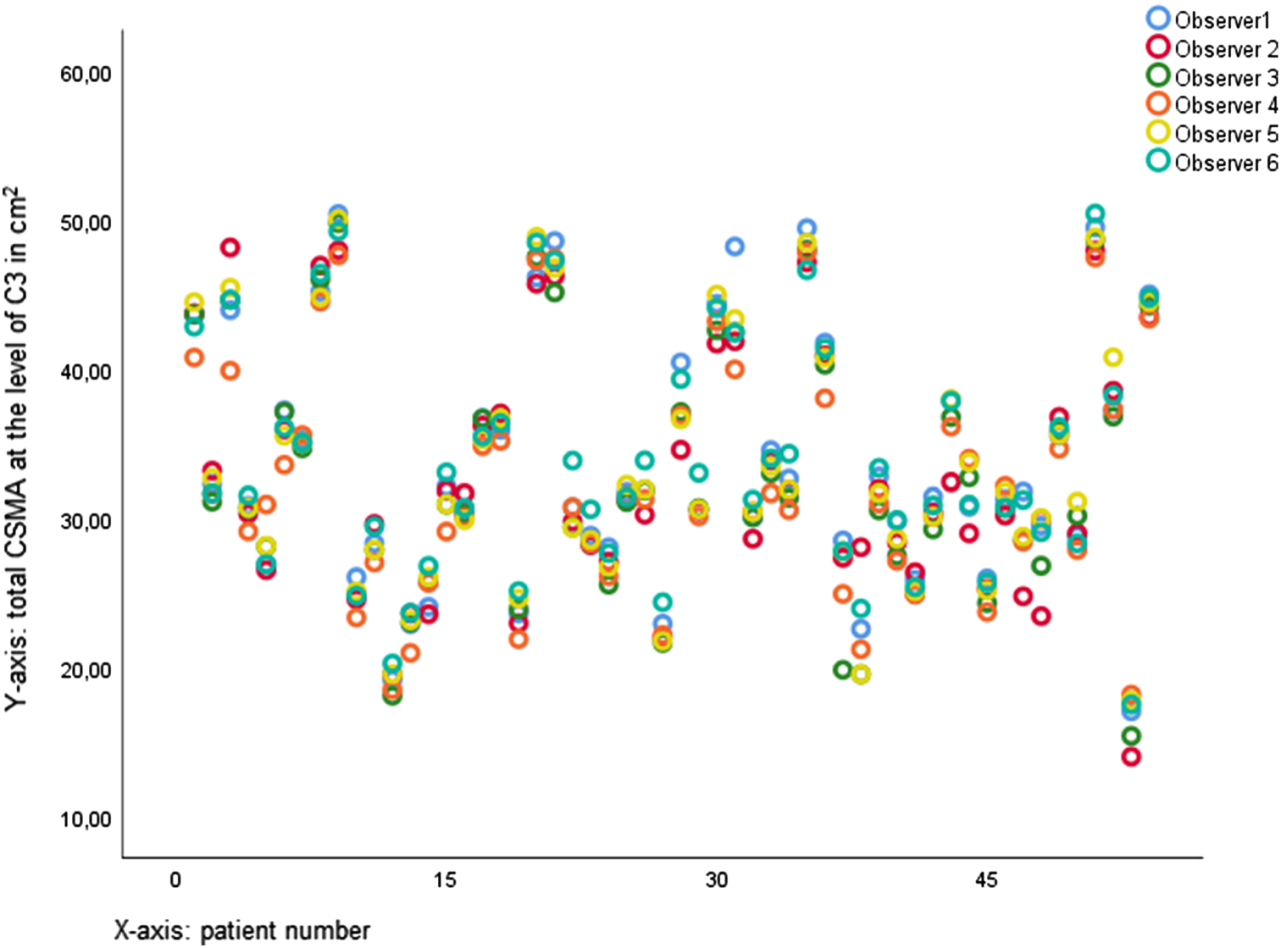
Intra-class correlation plot for measurement of total CSMA measurement at C3. The correspondence between measurements is visualized in the intra-class correlation plot

**Fig. 3:**
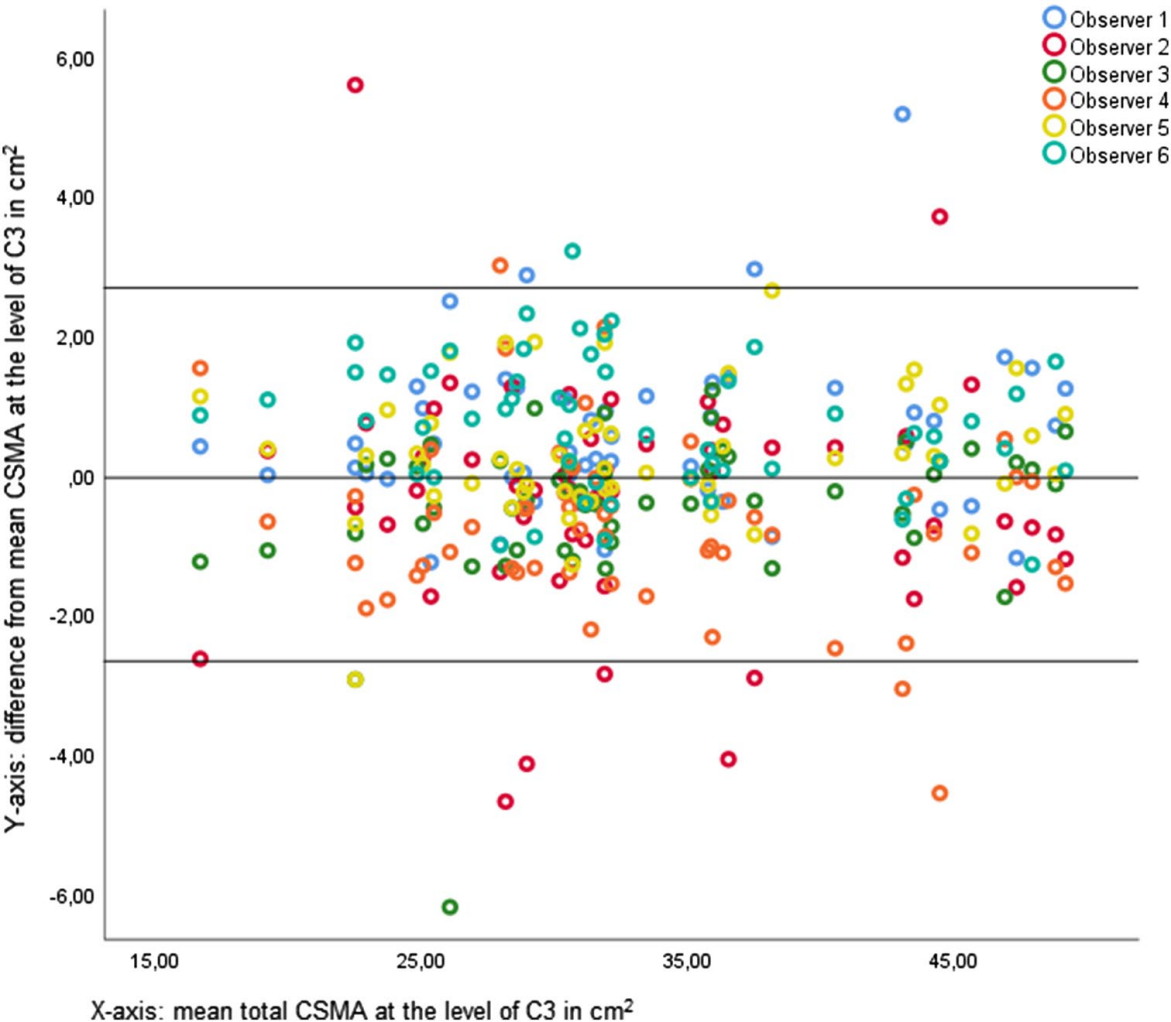
Bland–Altman plot for agreement of total CSMA measurement at C3 between all observers. Bland–Altman plots showing agreement between measurements of observers. The solid lines depict the mean and 95% limits of agreement in the Bland–Altman plot

Mean CSMA of the PVM, left SCM, and right SCM and total CSMA at C3 are shown in Table 3. Most CSMA measurements were normally distributed (Shapiro–Wilk: *p* > 0.05). There was a significant difference between the observers in CSMA measurements (repeated measures ANOVA: *p* < 0.001). Actual differences between CSMA measurements of all observers were small; the largest mean difference between observers was 1.48 cm^2^ (observer 6–observer 4). For all CSMA measurements, ICCs were excellent (0.763–0.969; all *p* < 0.001), showing good conformity between measurements of different observers, as visualized in Fig. 2. Figure 3 shows a combined Bland–Altman plot of total CSMA at C3 measurements of the individual observers and their difference to the overall mean CSMA at C3 measurements. The mean of the standard deviation of the difference between observer CSMA measurements and mean CSMA measurement was used to calculate the limits of agreement (95% confidence interval). There appears to be an element of systemic bias in CSMA measurement at C3, with some observers having more than 5% measurements outside of the 95% limits of agreement, and some observers systemically deviate one way (higher or lower) from the mean CSMA measurement. Actual differences are small.

**Table 3:** Cross-sectional muscle area (CSA) at the level of C3

	Total (*n* = 324)	Obs. 1 (*n* = 54)	Obs. 2 (*n* = 54)	Obs. 3 (*n* = 54)	Obs. 4 (*n* = 54)	Obs. 5 (*n* = 54)	Obs. 6 (*n* = 54)	*p* value*	ICC (95% CI)
CSMA PVM	28.03 (6.63)	28.52 (6.84)	27.95 (6.52)	27.45 (6.83)	27.53 (6.56)	28.15 (6.67)	28.59 (6.59)	< 0.001	0.969 (0.953–0.981)
CSMA left SCM	2.62 (1.30)	2.71 (1.35)	2.56 (1.40)	2.61 (1.25)	2.47 (1.22)	2.68 (1.30)	2.70 (1.32)	< 0.001	0.821 (0.754–0.880)
CSMA right SCM	2.44 (1.34)	2.43 (1.46)	2.22 (1.62)	2.58 (1.18)	2.33 (1.24)	2.54 (1.23)	2.53 (1.32)	< 0.001	0.763 (0.680–0.837)
Total CSMA at C3	33.09 (8.07)	33.66 (8.28)	32.73 (8.13)	32.64 (8.40)	32.34 (7.80)	33.37 (8.22)	33.82 (7.82)	< 0.001	0.969 (0.952–0.980)

All values are represented as mean (SD)

*CSA* cross-sectional muscle area, *PVM* paravertebral muscles, *SCM* sternocleidomastoid muscle, *Obs* observer

*p value calculated using a repeated measures ANOVA with a Greenhouse–Geisser correction for sphericity

## Discussion

This study aimed to examine the robustness of the C3 measurement method for SMM in terms of interobserver agreement and specific vertebra and single slice selection by multiple observers. This study was performed without a training data set and limited formal training, to simulate the use of the C3 measurement method for SMM as if it were adopted from an external research paper.

The interobserver agreement for all different CSMA measurements was excellent. The measurement of CSMA of PVM appears to be most uniform. There is some difference between observers in measurement of the CSMA of the SCM muscles; however, actual differences remain small. The previous research has shown that total CSMA at C3 was significantly better correlated with CSMA at L3 than the CSMA of the PVM only with CSMA at L3 [20].

The level of C3 is easily identifiable for both researchers and clinicians, with only incidental selection of a different level. There was more variation in the actual single slice selection, perhaps due to the lack of a training data set and little informal training received prior to delineation of scans. This may also be due to the thin slices (1 mm) and small (2 mm) interslice gaps of the CT imaging used in this study. In most cases, where an observer had selected a different slice than the majority of the observers, the different slices were indeed directly above or below of the slice the majority chose, which corresponds with a 3 mm difference in location. Probably, in these cases, the accidental selection of a different slice should not greatly influence CSMA measurements. However, it may still be advisable to include a training data set when starting to use the C3 measurement method to allow for a learning period in delineation of skeletal muscle and single slice selection.

In recent years, SMM has widely been researched in cancer patients, using a measurement of skeletal muscle area at the level of L3 on CT imaging as an indicator of total SMM [11]. Imaging at the level of L3 is not routinely performed in HNC patients, so SMM measurement at the level of L3 is not always clinically applicable in HNC [19]. The SMM measurement method at the level of C3 provides a reliable and robust alternative to SMM measurement at the level of L3, allowing for broad research into the predictive and prognostic effect of sarcopenia in HNC.

Patients with HNC often present with signs of malnutrition and as such are at risk of developing sarcopenia [29]. Adverse outcomes associated with sarcopenia, such as chemotherapy-related toxicity and wound healing problems, are highly prevalent in HNC [30]. It can be anticipated that both are at least partly related to sarcopenia. The first study using the C3 measurement method to assess SMM in HNC patients undergoing chemoradiotherapy found that low SMM was an independent predictor of the occurrence of chemotherapy dose-limiting toxicity [21]. A recent study in HNC patients undergoing laryngectomy for laryngeal cancer showed that low SMM, as measured at the level of L3, was an independent predictor of the occurrence of a pharyngocutaneous fistula and of the occurrence of any wound complication [31]. In this study, 122 patients who had undergone a total laryngectomy were retrospectively evaluated for inclusion; 70 (57%) had abdominal imaging available for analysis. It is likely that all or almost all HNC patients will undergo CT or MRI imaging of the head and neck area during the diagnostic process and follow-up [19]. The C3 measurement method allows the investigation of body composition in nearly all HNC patients without the need for extra diagnostics. A recent study in patients undergoing laryngectomy, where SMM was measured at the level of C3, showed that low SMM was a predictor of the occurrence of a pharyngocutaneous fistula [23]. Preoperative low SMM also was a strong negative prognostic factor for overall survival after laryngectomy [23]. In this retrospective study, 235 out of 245 patients could be included for analysis due to measurement of SMM at the level of C3.

Future research is still needed to clarify whether the adverse effects of low SMM are prognostic only, or if these adverse effects can be overturned. In the future, HNC patients at high risk of adverse outcomes related to low SMM or sarcopenia might benefit from additional supportive treatment or individualized primary treatment. Several possible interventions may be considered, such as altered chemotherapy dosing [32], prehabilitation before surgery [33], enhanced recovery after surgery [34], intensive physiotherapy [35], and additional nutritional support [36]. A measurement of SMM at the level of C3 might be used as a screening tool for low SMM in HNC cancer patients at diagnosis.

## Limitations

There are some limitations to this study that need to be addressed. No training data set or formal training was given to the observers in this study. Although this study setup was chosen, because it may best mimic the clinical adaptation and use of the C3 measurement method by other institutions, it may also explain some of the differences in the measurements and in the vertebra and single slice selections. For further use in research and clinical work, a training data set for researchers learning skeletal muscle area measurement may limit these differences. Second, only CT imaging was used in this study to assess SMM, as is usually done in other studies in cancer patients that assess SMM at the level of L3. Some HNC patients will only undergo MRI imaging of the head and neck area during the diagnostic process. It should still be evaluated whether CT and MRI can be used interchangeably for SMM assessment or that some form of modification of the method is necessary. Finally, most variation in CSA measurements was seen in the SCM muscles. This can at least partially be explained by lymph node metastasis close to or invading in the SCM muscles. Most patients included in this study had advanced, lymph node positive disease, which may make accurate delineation of the muscle more difficult. In daily clinical practice, approximately two-thirds of HNC patients present with advanced stage disease (large tumor and/or lymph node positive) [37]. Thus, this study population provides an accurate reflection of the clinical use of the C3 measurement method. Because previous research showed that the addition of the CSA of the SCM muscles was beneficial for a prediction model, and because actual differences were small and ICCs could still be classified as excellent, it is justified to include the CSA of the SCM muscles in the total CSA at C3 in patients with lymph node positive disease.

## Conclusion

Interobserver agreement is excellent for SMM measurement on head and neck CT imaging at the level of C3, as tested in a setting that mimics a clinical setting. The C3 measurement method for SMM is robust, easy to use and can be done on routinely performed CT imaging of the head and neck area. It allows for retrospective and prospective research into the predictive and prognostic value of low SMM in the vast majority of HNC patients, as well as use in possible future trials.

## Abbreviations

SMM: Skeletal muscle mass
HNC: Head and neck cancer
C3: Third cervical vertebra
L3: Third lumbar vertebra
CSMA: Cross-sectional muscle area
HU: Hounsfield unit
Lumbar SMI: Lumbar skeletal muscle index
